# Postprandial Inflammatory Stress: A Hypothesis-Generating Framework Linking Metabolic, Immune and Vascular Responses

**DOI:** 10.3390/life16081298

**Published:** 2026-08-07

**Authors:** Roko Šantić, Marko Kumrić, Lovre Martinović, Nikola Pavlović, Azer Rizikalo, Marino Vilović, Josip Vrdoljak, Joško Božić

**Affiliations:** 1 Department of Pathophysiology, University of Split School of Medicine, 21000 Split, Croatia; roko.santic@mefst.hr (R.Š.); marko.kumric@mefst.hr (M.K.); lovre.martinovic@mefst.hr (L.M.); nikola.pavlovic@mefst.hr (N.P.); marino.vilovic@mefst.hr (M.V.); josip.vrdoljak@mefst.hr (J.V.); 2 Laboratory for Cardiometabolic Research, University of Split School of Medicine, 21000 Split, Croatia; 3 Department of Anatomy, School of Medicine, University of Mostar, 88000 Mostar, Bosnia and Herzegovina; azer.rizikalo@mef.sum.ba

**Keywords:** postprandial inflammation, cardiometabolic disease, metabolic challenge, hypothesis-generating framework, PRISM-CM (Postprandial Inflammatory Stress Modules)

## Abstract

Chronic low-grade inflammation accompanies cardiometabolic disease, while routine risk assessment is based mainly on fasting measurements. This structured narrative review summarises human evidence for discrete postprandial changes in triglyceride-rich lipoproteins and remnants, glucose and insulin, endotoxin-related markers, innate immune cells and endothelial function. The evidence is comparatively consistent for postprandial lipaemia, glycaemic excursions and acute flow-mediated dilation responses, whereas endotoxin, cytokine and cellular findings are smaller, assay-sensitive and less consistently replicated. Based on this evidence, we introduce PRISM-CM (Postprandial Inflammatory Stress Modules in CardioMetabolic disease) as an author-developed, hypothesis-generating evidence map. It comprises five candidate biological domains—lipid–remnant burden, glucose–insulin stress, endotoxin handling, innate immune-cell activation and endothelial response—with recovery kinetics treated as a cross-domain analytic dimension. The framework was not derived by clustering, consensus methods or predictive modelling; its illustrative response patterns are not validated endotypes. A composite score is not proposed because the independence, reproducibility and incremental predictive value of the candidate measurements have not been established. Potential future diagnostic and prognostic applications require prospective validation. Reference ranges, age- and sex-specific norms, within-person reproducibility, reproducible response patterns and outcome-linked thresholds remain unknown. PRISM-CM is therefore not a clinical algorithm, risk score or treatment-selection tool.

## 1. Introduction

Chronic low-grade inflammation is now widely viewed as a mechanistic bridge between metabolic imbalance—such as insulin resistance, ectopic lipid accumulation, dysglycaemia, and metabolic dysfunction-associated steatotic liver disease (MASLD/MAFLD)—and progressive vascular injury culminating in atherothrombotic events [1,2,3]. Yet the biomarkers that clinicians routinely use to stratify this risk almost exclusively involve fasting: LDL-C, fasting triglycerides, fasting glucose, glycated haemoglobin (HbA1c) and hsCRP. These measurements describe the resting set-point of the cardiometabolic system. They say comparatively little about how that system behaves when challenged.

This matters because the human body is rarely fasting. Three to five eating occasions per day, each followed by a multi-hour absorptive and post-absorptive transition, mean that most adults spend the bulk of their day in some phase of postprandial metabolism [4,5,6,7]. During each transition, intestinal absorption of fats and carbohydrates floods the circulation with TRL, free fatty acids, glucose and gut-derived signals such as lipopolysaccharide (LPS), bile acids, and incretins [8]. The vasculature is exposed to a transient prothrombotic, pro-oxidant and pro-inflammatory milieu, with measurable reductions in FMD and increases in monocyte activation, soluble adhesion molecules and selected cytokines [9,10,11,12,13].

Large prospective epidemiological studies have shown that nonfasting triglycerides predict cardiovascular events at least as well as fasting values, and that nonfasting remnant cholesterol carries independent atherogenic risk beyond LDL-C [14,15,16,17,18,19]. These findings provide population-level rationale for measuring lipids in the nonfasting or postprandial state. They do not, however, capture the dynamic dimension of postprandial physiology: the magnitude of biomarker excursion, the timing of peaks, the rate of recovery and the integration across multiple biological axes within a single individual.

The evidence reviewed below supports several discrete observations: nutrient challenges can produce postprandial lipaemia and glycaemic excursions, alter selected immune and endotoxin-related measures, and transiently impair endothelial function in some settings. The authors’ separate conceptual proposal is that these measurements could be studied concurrently as PRISM-CM (Postprandial Inflammatory Stress Modules in CardioMetabolic disease). PRISM-CM is intended to generate testable hypotheses; it does not establish a latent syndrome, stable response endotypes, superior risk prediction or treatment relevance. Any potential future diagnostic and prognostic application requires prospective validation.

## 2. Materials and Methods

### 2.1. Literature Identification, Eligibility and Appraisal

This article is a structured narrative review and author-led conceptual synthesis rather than a systematic review, meta-analysis or biomarker-derivation study. Database coverage extended from inception through 30 June 2026. PubMed/MEDLINE, Scopus and Web of Science were searched; Google Scholar was used for forward citation tracking, and reference lists of eligible articles and relevant reviews were examined manually. No database-level language filter was applied. Detailed appraisal was restricted to publications for which sufficient English-language information was available.

The primary PubMed/MEDLINE syntax was: (“postprandial”[Title/Abstract] OR “oral fat tolerance”[Title/Abstract] OR “mixed meal”[Title/Abstract] OR “nonfasting”[Title/Abstract]) AND (inflamm*[Title/Abstract] OR endotox*[Title/Abstract] OR lipopolysaccharide[Title/Abstract] OR “endothelial function”[Title/Abstract] OR “flow-mediated dilation”[Title/Abstract] OR monocyte*[Title/Abstract] OR “remnant cholesterol”[Title/Abstract] OR “triglyceride-rich lipoprotein*”[Title/Abstract] OR “ApoB-48”[Title/Abstract] OR “apolipoprotein B-48”[Title/Abstract] OR “ApoC-III”[Title/Abstract] OR “apolipoprotein C-III”[Title/Abstract] OR glucose[Title/Abstract] OR insulin[Title/Abstract]). The equivalent Scopus search used TITLE-ABS-KEY and the Web of Science search used TS with the same concept blocks. Additional targeted searches combined these terms with obesity, insulin resistance, prediabetes, type 2 diabetes, metabolic syndrome, MASLD/MAFLD, atherosclerosis and cardiovascular risk; disease terms were not mandatory because this would exclude informative challenge studies in healthy participants.

Core evidence comprised: (i) original adult human studies using a mixed-meal, oral-fat, glucose or other controlled nutrient challenge with a baseline and at least one postchallenge measurement relevant to a candidate domain; (ii) prospective studies examining nonfasting triglycerides, remnant cholesterol or related markers in relation to vascular or cardiovascular outcomes; and (iii) randomised interventions reporting direct postprandial metabolic, inflammatory, cellular or vascular outcomes. Reviews, consensus papers and preclinical or in vitro studies were retained only for methodological or mechanistic context and were not treated as direct validation of the integrated framework. Publications were excluded from the core evidence map if they did not address postprandial or nonfasting physiology, reported no relevant outcome, duplicated another report without additional data, lacked sufficient methodological and outcome information for interpretation, or were conference abstracts or case reports. Paediatric studies were not used as primary evidence for the adult framework.

For core human studies, extracted characteristics included study design, sample size and population, challenge composition, sampling duration and timepoints, assay or functional method, principal findings and major limitations. Evidence was appraised qualitatively for directness, independent replication, sample size, phenotype definition, challenge and assay standardisation, kinetic completeness, and availability of prospective outcomes. These judgments are not formal GRADE ratings; no quantitative pooling or formal risk-of-bias assessment was performed. Particular caution was applied to endotoxin and LPS-related assays because matrix effects, lipoprotein binding, sample handling and inter-assay variability affect comparability.

Duplicate records were managed in Zotero using the Duplicate Items function and then checked manually by DOI, title, first author and publication year. Following the revision update, six publications falling within the stated search period and one relevant contextual review published after the database-search cutoff were added, yielding a final narrative corpus of 108 unique cited publications. This count is not described as 108 included studies because the corpus contains original studies, reviews and contextual sources. The original search was not prospectively logged as a systematic review; database-specific retrieval totals and counts excluded at each stage cannot be reconstructed reliably and were not estimated retrospectively. Supplementary Figure S1 therefore presents the documented evidence-identification and conceptual-synthesis workflow rather than a PRISMA flow diagram.

### 2.2. Construction and Interpretation of PRISM-CM

PRISM-CM was constructed through an author-led qualitative evidence-mapping process and was not derived statistically. A candidate biological domain was retained when it: (i) showed a measurable dynamic response after a nutrient challenge in original human research; (ii) represented a biologically distinct process plausibly linking nutrient exposure with inflammatory or vascular responses; (iii) was not fully redundant with another domain; (iv) permitted time-resolved characterisation; and (v) had potential feasibility in prospective research. Greater interpretive weight was given to replicated human evidence than to mechanistic or preclinical work. Selection was not based on citation frequency, a Delphi procedure, formal expert consensus, cluster analysis, latent-class analysis or predictive modelling.

The resulting map contains five candidate biological domains: lipid–remnant burden, glucose–insulin stress, endotoxin handling, innate immune-cell activation and endothelial response. Recovery kinetics are treated as a cross-domain analytic property derived from serial measurements rather than as a sixth independently validated biological module. Circulating cytokines are considered secondary, cross-domain measurements because their acute postprandial behaviour is inconsistent. The candidate response patterns introduced later are illustrative one-to-one extensions of these domains; they were not discovered empirically. No biomarker weighting, threshold, composite formula or treatment rule was derived.

**Figure 1:**
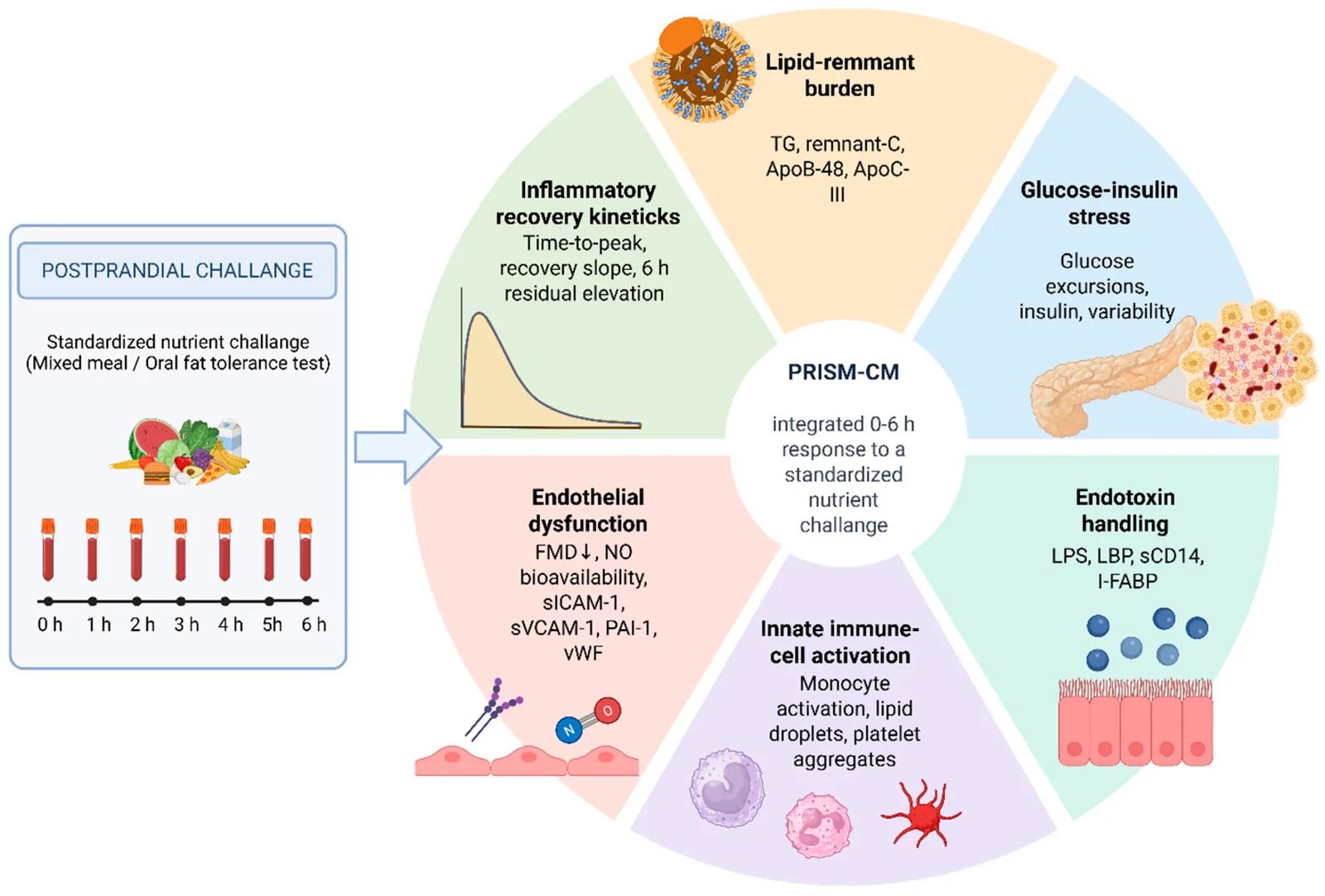
Author-proposed, hypothesis-generating PRISM-CM evidence map. A standardised postprandial nutrient challenge may be used to measure five candidate biological domains—lipid–remnant burden, glucose–insulin stress, endotoxin handling, innate immune-cell activation and endothelial response—while recovery kinetics are analysed across domains. The diagram is a conceptual synthesis and does not establish a causal sequence, validated classification, composite score or clinical algorithm. Abbreviations: ApoB-48, apolipoprotein B-48; ApoC-III, apolipoprotein C-III; FMD, flow-mediated dilation; I-FABP, intestinal fatty acid-binding protein; LBP, lipopolysaccharide-binding protein; LPS, lipopolysaccharide; NO, nitric oxide; PAI-1, plasminogen activator inhibitor-1; remnant-C, remnant cholesterol; sCD14, soluble CD14; sICAM-1, soluble intercellular adhesion molecule-1; sVCAM-1, soluble vascular cell adhesion molecule-1; TG, triglyceride; vWF, von Willebrand factor. Created in BioRender. Kumric, M. (2026) https://BioRender.com/wg3qmda.

Throughout the review, statements about individual postprandial phenomena are presented as evidence-supported observations when directly supported by human studies. Statements about concurrent integration, response patterns, recovery constructs and possible future applications are explicitly identified as the authors’ conceptual proposals. Supplementary Figure S1 summarises the review workflow, while Figure 1 depicts the hypothesis-generating PRISM-CM map.

## 3. The Postprandial State as a Cardiometabolic Stress Test

A standardised mixed-meal or oral fat tolerance test (OFTT) can be used as a physiological challenge in a manner conceptually analogous to the oral glucose tolerance test (OGTT) [7,20,21]. Unlike the glucose-focused OGTT, mixed-meal and fat challenges permit serial observation of lipid absorption and clearance together with glycaemic, immune, gut-derived and vascular measurements. PRISM-CM proposes studying selected measurements concurrently; it does not alter the established purpose of the underlying tests.

Serial measurement distinguishes fasting level from response magnitude, timing and recovery. Individuals with similar fasting values can show different postchallenge trajectories, and mean curves can obscure inter-individual heterogeneity [6,7,20,22]. However, heterogeneous trajectories do not by themselves establish stable biological endotypes. In this review, “delayed recovery” denotes only a candidate kinetic descriptor that requires a prespecified mathematical definition and repeatability testing.

Candidate kinetic readouts include peak concentration, incremental and total area under the curve, time to peak, recovery slope, and residual elevation at the final timepoint. Their relevance depends on the biomarker and challenge duration; none currently has a PRISM-CM threshold. Historical work proposed postprandial chylomicron remnants as atherogenic [4,5], quantified remnant kinetics in humans [23,24] and demonstrated postprandial activation of coagulation factor VII [25]. These observations establish component phenomena, but they do not demonstrate a coordinated multi-domain phenotype.

### Relationship to Established Postprandial and Vascular Tests

PRISM-CM is not proposed as a replacement for opportunistic nonfasting triglyceride testing, OGTT, mixed-meal tolerance testing, OFTT or flow-mediated dilation (FMD). Nonfasting triglycerides provide pragmatic population-level risk information; OGTT has established diagnostic thresholds; mixed-meal and OFTT protocols characterise nutrient handling; and FMD is an operator-dependent research measure of vascular function. These antecedent approaches are better standardised and more mature than PRISM-CM. Contemporary reviews also show substantial heterogeneity in meal composition and sparse endothelial assessment across acute high-fat studies [26].

The narrower novelty proposed by the authors is the concurrent, time-resolved mapping of selected metabolic, gut-derived, cellular and vascular measurements after the same challenge. Whether such integration is reproducible or adds information beyond established tests is unknown and must be evaluated prospectively.

## 4. Evidence Relevant to Candidate PRISM-CM Domains

This section distinguishes observations supported for individual postprandial processes from the authors’ proposal to place them in a common framework. Evidence supporting one domain does not validate PRISM-CM as an integrated construct, and evidential maturity differs substantially across domains.

### 4.1. Triglyceride-Rich Lipoproteins, Remnant Cholesterol and ApoB-48 as Postprandial Inflammatory Triggers

Postprandial triglycerides remain the most accessible lipid readout of a fat challenge, but they are an incomplete marker of atherogenic exposure. The bulk of cardiovascular risk attributable to nonfasting hypertriglyceridaemia appears to reflect the cholesterol carried in TRL remnants—chylomicron remnants of intestinal origin and very-low-density lipoprotein (VLDL) remnants of hepatic origin—rather than triglyceride molecules themselves [15,16,17,18,27]. Remnant cholesterol, ApoB-48 (a specific marker of intestinally derived particles) and apolipoprotein C-III (ApoC-III, a regulator of TRL clearance) collectively capture the atherogenic remnant burden more directly than total triglycerides [23,24,28].

Observational cohorts and Mendelian-randomisation analyses associate remnant cholesterol with ischaemic heart disease and low-grade inflammation independently of LDL-C [18,19], while major cohorts show that nonfasting triglycerides predict cardiovascular events at least as well as fasting values [14,15,16]. These findings support population-level relevance of remnant exposure but do not establish that an integrated postprandial panel improves prediction. A recent trial-level systematic review and meta-regression found no independent association between remnant-cholesterol reduction and cardiovascular outcomes after accounting for correlated lipid changes, providing an important caution against overinterpreting causality from biomarker associations [29].

In a 6-year follow-up of 107 adults with normal fasting lipid profiles, postprandial hypertriglyceridaemia in an OFTT predicted carotid atherosclerosis despite indistinguishable fasting lipids at baseline [30]—precisely the population that fasting algorithms most reliably miss. Mechanistically, postprandial TRLs infiltrate the arterial wall, are taken up by macrophages and monocyte-derived cells, and act as substrates for foam-cell formation [7,31]. They also engage circulating monocytes directly: in healthy adults fed a standardised high-fat meal, monocyte CD11c expression rose at 3.5 h postprandially, correlated with triglycerides, and was accompanied by increased monocyte lipid uptake and enhanced adhesion to vascular cell adhesion molecule-1 (VCAM-1) [32]. VLDL lipolysis products promote monocyte activation in vitro and in vivo [27].

Biological modifiability of the lipid–immune axis is illustrated by a small mechanistic study in which ApoC-III inhibition reduced postprandial TRL accumulation in monocytes and attenuated selected inflammatory changes [33]. Phase 3 olezarsen trials demonstrate robust lowering of triglyceride-rich lipoprotein burden in defined hypertriglyceridaemic populations [34,35]. These findings do not validate PRISM-CM response patterns, show incremental prediction or establish phenotype-matched therapy. The author-proposed contribution is therefore limited to testing whether lipid, immune and vascular responses covary after a common challenge.

### 4.2. Gut-Derived Inflammatory Signals and Endotoxin Handling

A high-fat meal does more than raise plasma lipids. Chylomicron assembly within enterocytes is accompanied by the apparent uptake and transport of small amounts of bacterial LPS from the gut lumen [3,6,36,37]. In a controlled study, healthy men consuming a 50 g fat meal showed acute elevations in plasma endotoxin, providing direct human evidence for a postprandial endotoxaemia phenomenon [37]. Subsequent work in lean and obese men using lipid dose-effect designs showed that postprandial endotoxin appearance is tied to chylomicron formation and differs between adiposity categories, supporting a chylomicron-mediated transport model [38,39].

The preclinical rationale comes from murine studies in which high-fat feeding raised plasma LPS and recapitulated insulin resistance and hepatic inflammation [36], and from rat studies showing that meal-composition modulation can attenuate postprandial systemic oxidative stress and vascular endothelial dysfunction [40]; in humans, low-dose intravenous LPS infusion reproduced features of insulin resistance and dyslipidaemia, supporting causal plausibility [41]. Endotoxin itself is typically measured by Limulus amoebocyte lysate (LAL) assay or endotoxin activity assay (EAA); LPS-binding protein (LBP) and soluble CD14 (sCD14) chaperone LPS and integrate exposure over hours to days [39,42]; intestinal fatty acid binding protein (I-FABP) marks small-intestinal barrier perturbation [43].

Population evidence links endotoxaemia to cardiometabolic disease. In Finnish cohorts, baseline endotoxaemia was associated with incident T2D and adverse cardiovascular outcomes [44,45,46]. In morbidly obese adults, the postprandial endotoxin rise after oral fat overload tracked postprandial hypertriglyceridaemia, coupling LPS handling to TRL kinetics [47]. T2D patients show a more pronounced postprandial endotoxin response than controls [48], and postprandial LBP and sCD14 trajectories differ by adiposity [39]. In pregnancy, markers of intestinal permeability such as serum zonulin have been associated with metabolic risk in overweight women [49], suggesting that barrier-function phenotypes may be relevant beyond standard cardiometabolic cohorts [50]; however, this should not be interpreted as direct evidence of a postprandial LBP phenotype.

Three caveats must be stated explicitly. First, LPS quantification is methodologically challenging: matrix interference, lipoprotein binding, sample handling and inter-laboratory assay variability all influence absolute values, and standardisation remains imperfect [42,51]. Second, not every study confirms a robust postprandial LPS rise; in a careful analysis of single high-fat and moderately high-fat meals in healthy adults, postprandial inflammation occurred without consistent endotoxaemia, prompting a re-examination of the LPS-centric model [51]. Toll-like receptor 4 (TLR4) activation by saturated fatty acids has also been shown to depend on LPS contamination rather than direct lipid signalling [37]. Third, dietary fat quality and emulsification influence the postprandial endotoxin signal more than fat quantity alone [52,53,54,55,56]. We therefore treat LPS, LBP, sCD14 and I-FABP as a coordinated “endotoxin-handling module”—biologically plausible and promising for research, but not a single validated clinical biomarker.

### 4.3. Innate Immune-Cell Activation After Postprandial Metabolic Stress

Circulating monocytes are well placed to integrate postprandial lipid, glycaemic and gut-derived signals. They express receptors for oxidised and remnant lipoproteins, toll-like receptors that respond to LPS and saturated lipids, and adhesion molecules that govern their interaction with the endothelium. Postprandial monocyte activation can therefore be considered a functional readout of integrated cardiometabolic stress rather than a parallel reading [27,32,57].

Monocyte CD11c and CD11b expression rises during postprandial hypertriglyceridaemia and tracks the lipid excursion [32]. Monocyte lipid droplet content increases postprandially, reflecting uptake of TRL-derived lipid [32,33]. Monocyte–platelet aggregates rise after a high-fat meal in normolipaemic men [58], and postprandial monocytes show enhanced endothelial adhesion and transmigration ex vivo [32]. Monocyte subset distribution (classical CD14++CD16−, intermediate CD14++CD16+, non-classical CD14+CD16++) adds granularity, with intermediate monocytes linked to atherothrombosis [57].

Interventional and meal-challenge studies provide mechanistic support. ApoC-III inhibition reduced monocyte lipid accumulation and selected activation markers [33], while a high-fat/high-carbohydrate meal increased peripheral-mononuclear-cell TLR2, TLR4 and SOCS-3 together with endotoxin-related measures [59,60]. Tissue-factor-positive monocyte-derived microparticles may contribute to transient prothrombotic activity, whereas human postprandial evidence for neutrophil activation, NETosis and complement engagement remains sparse. The authors therefore propose immune-cell activation as a candidate research domain. No current evidence demonstrates that these measurements are more reproducible or prognostic than circulating cytokines, or that they add predictive value to standard tests.

### 4.4. Endothelial Activation and Vascular Dysfunction as Downstream Readouts

The endothelium is a plausible downstream tissue in which postprandial lipid, immune and oxidative signals may converge [1,61,62]. Brachial artery FMD is an established research measure and predicts incident cardiovascular events in prospective cohorts [63]. Small acute studies reported transient FMD impairment after high-fat meals [9], including the pioneering 900-kcal study [9], attenuation by antioxidant vitamins [10], associations with triglyceride excursions [11] and greater endothelial activation in type 2 diabetes [12,64]. Nevertheless, a recent systematic review identified only two studies assessing endothelial function among 16 eligible acute low-carbohydrate, high-fat meal trials, underscoring the limited and heterogeneous evidence base.

Soluble endothelial biomarkers—namely, sE-selectin, sICAM-1, sVCAM-1, von Willebrand factor (vWF), thrombomodulin and plasminogen activator inhibitor-1 (PAI-1)—complement FMD but follow slower, more phenotype-dependent kinetics and are less consistently captured within a single 6 h meal-challenge window [12,52,65]. In an adiposity-stratified high-fat-meal study, women with obesity had higher soluble E-selectin, leptin and PAI-1 across timepoints, yet the meal itself did not significantly change circulating inflammatory or endothelial injury biomarkers within either group [65]. FMD typically shows clearer postprandial impairment within 6 h; soluble markers may better integrate chronic activation over days [66]. Tissue-factor-positive extracellular vesicles add a coagulation dimension relevant to obesity and metabolic syndrome [59,60,67].

Mechanistically, postprandial lipaemia drives oxidative stress, reduces nitric oxide bioavailability and leukocyte adhesion [9,10,11,12], and oscillating glucose may be more deleterious than steady hyperglycaemia for endothelial function [13]. The response is modifiable: a single bout of prior high-intensity aerobic interval exercise fully prevented postprandial FMD impairment in healthy adults [68], a single aerobic bout the morning of the meal attenuated postprandial responses in overweight/obese adults [69], and acute aerobic exercise blunted high-fat-meal-induced FMD impairment in young men [44]. Diet composition matters: replacing carbohydrate energy with avocado improved postprandial glycaemic, insulinemic and FMD responses in overweight/obese adults [70]; avocado added to a hamburger meal modulated vascular reactivity and postprandial inflammation [71]; a walnut-rich meal preserved FMD better than an isocaloric olive-oil meal [72]; and walnut consumption acutely improved postprandial FMD in hypercholesterolaemic adults [73]. Longer-term Mediterranean-style dietary interventions improve conventional cardiovascular risk factors and, in separate trials, endothelial function and vascular inflammatory markers in metabolic syndrome, whereas acute postprandial endothelial effects are better supported by meal-component studies using olive oil, walnuts, avocado or other Mediterranean-diet components [70,71,72,73,74,75]. Conversely, brief passive smoking can acutely impair endothelial function via oxidative stress, so smoking and passive smoke exposure should be treated as important confounders in postprandial vascular studies [76].

### 4.5. Cytokines and Acute-Phase Biomarkers: Useful but Insufficient Alone

Interleukin-6 (IL-6), IL-1β, IL-18, tumour necrosis factor-α (TNF-α), monocyte chemoattractant protein-1 (MCP-1) and hsCRP are familiar inflammatory biomarkers, but their acute postprandial behaviour is heterogeneous. Acute experimental hyperglycaemia increased several cytokines [13], whereas mixed-meal and high-fat challenges frequently produce small, variable or assay-sensitive changes [51,53,54,77]. In a 2026 double-blind four-way crossover trial of 18 participants that profiled 93 inflammatory proteins, meals differing in fatty-acid source did not produce a coherent directional inflammatory signature [78].

hsCRP is more informative as a chronic risk marker than as an acute single-meal signal because hepatic synthesis follows a longer time course. Chronic cohort evidence supports inflammation as a component of cardiometabolic risk [66]; lipid lowering has reduced events in patients selected for elevated hsCRP [79]; and anti-inflammatory intervention has reduced cardiovascular events in selected patients [2,80]. That chronic evidence does not validate transient meal-related cytokine changes or their integration into PRISM-CM. Cytokines are therefore treated here as secondary, cross-domain measurements rather than a separate module or required component.

### 4.6. Glucose–Insulin Axis and Postprandial Vascular Stress

Postprandial hyperglycaemia is an independent contributor to vascular injury in T2D and prediabetes [81,82,83,84,85,86]; the DECODE programme and related studies linked 2 h postchallenge glucose to cardiovascular mortality more tightly than fasting glucose [81,82]. Mechanistically, glucose excursions drive oxidative stress, advanced glycation end-product formation, mitochondrial reactive oxygen species and endothelial activation [15,33,39,40]; postprandial hyperglycaemia transiently impairs FMD in T2D [83]; and oscillating glucose may be more deleterious than steady hyperglycaemia [87].

Glycaemic and lipaemic excursions are partially independent. Acute human studies link postchallenge glucose exposure with oxidative stress and endothelial impairment [86], and oscillating glucose may be more deleterious than sustained hyperglycaemia [87]. Postprandial hyperglycaemia has recognised clinical relevance beyond fasting glucose [82]. Recent mixed-meal data also demonstrate substantial heterogeneity and sex-related differences in glucose–insulin and triglyceride dynamics [88]. These observations support retaining glucose–insulin as a candidate domain, but no study has shown that its inclusion in PRISM-CM adds value beyond OGTT, HbA1c, continuous glucose monitoring or established postchallenge glycaemia. Representative human evidence informing the candidate PRISM-CM domains, together with the principal limitations of their integration, is summarised in Table 1.

## 5. Hypothesis-Generating Integration of Candidate Domains

### 5.1. Illustrative Postprandial Response Patterns

The authors propose six illustrative response patterns as hypotheses for future testing: lipid–remnant-dominant [15,16,18,19,23,28,30], endotoxin-handling-predominant [38,39,42,43,47], immune-cell-activation-predominant [27,32,33,57,58], endothelial-response-predominant [9,10,11,12,61,63], glucose–insulin-predominant [13,82,83,84], and delayed cross-domain recovery [53,60,82]. These labels are descriptive extensions of the candidate domains and are not empirically discovered clusters, stable endotypes or diagnostic categories. They may overlap, particularly in type 2 diabetes and metabolic syndrome [12,17,64,92]. Demonstrating their existence would require repeated standardised challenges, prespecified unsupervised or model-based analyses, assessment of within-person stability, external replication and association with outcomes. Until then, the labels should be used only to formulate research questions.

### 5.2. Requirements Before Development of a Composite Measure

Although individual studies support measurement of lipid/remnant [18,19,23,28,30], glucose–insulin [13,83,84,87], endotoxin-related [39,42,47], immune-cell [32,33,57,58], vascular [9,10,11,12,61,63], cytokine [51,77] and kinetic [30,53,82] signals, the available evidence does not justify proposing a numerical PRISM-CM score. No prospective dataset has shown that the candidate biomarkers contribute independently to prediction, cluster into reproducible response phenotypes, or improve discrimination, calibration or clinical utility beyond established fasting and nonfasting measures. A 2025 multi-study feasibility analysis further showed that a minimal postprandial cytokine composite failed in both datasets and that a broader composite was significant in only one [90]. PRISM-CM therefore denotes candidate measurement domains, not a score.

Any future composite development should proceed only after analytical and within-person reproducibility studies; assessment of dimensionality, redundancy and collinearity; prespecified derivation of weights and thresholds in adequately sized cohorts; comparison with established risk models, nonfasting lipids, OGTT and FMD; and internal and external validation across age, sex, ethnicity, metabolic phenotype and challenge protocol. Evaluation should include vascular imaging and event outcomes [30,61,63], with transparent reporting of calibration, discrimination and clinical utility. Until those steps are completed, no biomarker combination, cutoff, simplified clinical version or treatment-selection rule should be proposed. Table 2 separates measurements directly reported in human studies from the authors’ proposed uses within PRISM-CM.

## 6. Potential Research and Translational Applications

At present, PRISM-CM cannot be used for clinical risk stratification. Its candidate domains may be studied in research participants with central adiposity, metabolic syndrome, MASLD/MAFLD, prediabetes or a family history of premature cardiovascular disease, including those with unremarkable fasting measurements. Individual studies suggest that controlled challenges can reveal lipid, FMD or monocyte responses not evident from fasting values [30,32,33], and residual lipid or inflammatory risk is recognised in conventional settings [2,28,79,80]. Population-level associations support the relevance of nonfasting lipids [15,16,17,18,19,28]. However, no study has shown that concurrent PRISM-CM measurement improves prediction, reclassification or clinical outcomes beyond standard nonfasting triglycerides, remnant cholesterol, OGTT, HbA1c or established vascular assessments.

### 6.1. Diet and Lifestyle

Diet composition—including substitution of refined carbohydrate with fibre-rich, monounsaturated and polyunsaturated foods—improves postprandial glycaemic and lipemic responses [52,54,70,71,72,73,93]. Longer-term Mediterranean-style dietary patterns improve conventional cardiovascular risk factors, endothelial function and markers of vascular inflammation in metabolic syndrome [75,94], with acute meal-level effects supported by component studies [70,71,72,73,74]. Modest weight loss attenuates postprandial lipaemia [20,92]. Exercise, particularly as a prior acute bout and in combination with lipid-lowering therapy, attenuates postprandial lipaemia, protects FMD and may reduce selected postprandial inflammatory signals [44,68,69,89].

### 6.2. Pharmacological Modulation of Individual Postprandial Processes

Several pharmacological classes modulate postprandial cardiometabolic physiology. Statins reduce overall TRLs and remnant burden and their use is associated with lower postprandial lipid stress; adding exercise to statin therapy further lowers postprandial triglycerides in overweight individuals with hypercholesterolaemia [89,95]. Fibrates and high-dose icosapent ethyl (eicosapentaenoic acid, EPA) lower postprandial triglycerides and the latter reduces cardiovascular events on a statin background, although the mechanism is not exhausted by triglyceride-lowering [96,97]. Fenofibrate has been shown to improve postprandial endothelial function and reduce cell adhesion molecules during post-prandial lipaemia in hypertriglyceridaemic patients [97]. GLP-1 receptor agonists can slow early gastric emptying and improve postprandial glycaemic and lipid responses; human studies with exenatide, liraglutide, lixisenatide and semaglutide support reductions in intestinal lipoprotein production, postprandial triglyceride excursions, ApoB-48 or chylomicron–triglyceride handling [98,99,100,101]. SGLT2 inhibitors reduce glycaemic exposure through glucosuria and may improve selected postprandial or CGM-derived glucose measures in some populations, although the effect on glycaemic variability is not uniform across trials [102,103]. Evidence for vascular or inflammatory modulation by SGLT2 inhibitors is stronger for chronic biomarker and endothelial-function studies than for direct postprandial inflammatory challenge endpoints [104,105]. These mechanisms are biologically aligned with PRISM-CM modules but should be considered mechanistically plausible rather than validated phenotype-matched interventions.

ApoC-III–targeting strategies reduce ApoC-III and triglyceride-rich lipoprotein burden in phase 3 trials [34,35], and a small mechanistic study suggests effects on postprandial monocyte lipid accumulation and selected activation markers [33]. These findings support biological plausibility but not PRISM-CM-guided treatment selection. Older statin work also reports attenuation of postprandial lipaemia [95]. Whether any intervention improves a prespecified recovery metric or modifies outcomes specifically in an author-proposed response pattern remains an untested trial hypothesis.

### 6.3. T2D, Metabolic Syndrome and MASLD/MAFLD

Type 2 diabetes and metabolic syndrome are associated with altered postprandial handling, including reported changes in endotoxin-related measures [48], endothelial responses [12,64], FMD recovery [64] and remnant kinetics [24]. Postprandial dysmetabolism is a recognised component of cardiometabolic risk [7,17,81,92,106], and hepatic insulin resistance may alter lipid handling in MASLD/MAFLD [6,7,28,92]. These observations justify targeted research in these populations; they do not establish PRISM-CM as a clinical phenotyping tool.

## 7. Methodological Recommendations for Future Studies

Future studies should standardise the prechallenge state. Reports should specify clock time and phase relative to habitual sleep–wake timing; duration and quality of sleep on the preceding night; timing and composition of the previous meal; alcohol intake; smoking, vaping, nicotine and passive-smoke exposure; recent infection or acute illness; and all medications or supplements capable of altering lipid, glucose, vascular or inflammatory responses. Vigorous and moderate exercise should be restricted or standardised for 24–48 h, with the interval recorded. Menstrual-cycle phase, hormonal contraception, menopausal status and sex-specific analyses should be prespecified where applicable [76,89,95,97]. Identical meals administered at different times of day can yield different glucose and triglyceride responses, although small repeated-meal studies should not be interpreted as proof of an endogenous circadian mechanism [91]. More broadly, host circadian phase and time-varying microbial activity may shape interorgan postprandial glucose handling; however, causal dynamic coupling among the gut microbiome, liver, pancreatic islets, adipose tissue and skeletal muscle has not yet been demonstrated in humans [107].

Challenge protocols should report total energy, fat amount and fatty-acid distribution, carbohydrate type and load, protein, fibre, emulsification, and food matrix [52,53,54,77]. Sampling should include baseline and multiple postchallenge timepoints—commonly 1, 2, 3, 4 and 6 h—with extension to 8–10 h when delayed lipid clearance is central [7,30]. Kinetic outcomes should be prespecified for each biomarker, including peak, incremental and total AUC, time to peak, and a clearly defined late residual or recovery measure. For endotoxin-related outcomes, the assay platform, detection limit, sample handling, lipoprotein treatment and coefficients of variation should be reported [42,51]. Body position, premeasurement rest, ambient conditions and meal-consumption time should be standardised when vascular measurements are performed.

Where feasible, studies should combine soluble, cellular and functional measures rather than infer one domain from another [32,33,58]. Prospective cohorts should test analytical and within-person repeatability before examining vascular imaging or event outcomes [30,61,63,108]. Candidate patterns, clustering procedures and any future composite development must be prespecified, internally validated and externally replicated; post hoc labels should not be presented as established endotypes.

## 8. Limitations of the Postprandial Inflammatory Stress Framework

PRISM-CM has substantial limitations. It is an author-led conceptual map, not a statistically derived construct, and alternative domain structures are plausible. The candidate domains have not been shown to covary within individuals or to form reproducible response patterns. Reference ranges, age- and sex-specific norms, analytical and within-person reproducibility, thresholds, incremental predictive value, and outcome associations are not established. Many human studies are small, single-centre, phenotype-specific and based on surrogate endpoints; independent replication is uneven. Cytokine responses are variable [51,53,54,77] and endotoxin-related measurements remain assay-sensitive [42,51]. Recent systematic and experimental evidence reinforces heterogeneity in endothelial, cytokine and composite inflammatory responses [26,78,90]. A single postprandial timepoint is inadequate, but longer protocols increase burden and may reduce feasibility. Finally, this structured narrative review did not include a formal risk-of-bias assessment or quantitative synthesis, and stage-specific retrieval and exclusion counts were not prospectively recorded.

## 9. Future Perspectives

The first research priority is harmonisation of challenge composition, prechallenge conditions, sampling schedules and assay procedures [7,20,21,77]. The second is to establish analytical and within-person repeatability of each candidate measure, then test whether domains covary, whether any response patterns replicate and whether concurrent measurement adds information beyond established fasting and nonfasting tests. Only after those steps should adequately powered prospective cohorts examine vascular imaging and event outcomes [30,61,63,108]. Continuous glucose monitoring, nonfasting lipid testing and wearable sleep or activity measures may serve as contextual covariates or comparators rather than an implicit validation of PRISM-CM.

Intervention studies provide hypotheses rather than treatment recommendations. Future trials could test whether prespecified lipid/remnant responses modify the effects of lipid-lowering approaches [33,34,35,89,95,96]; whether endothelial responses are reproducibly altered by exercise or dietary interventions [44,68,69,75,94]; whether immune-cell measurements respond to ApoC-III inhibition [33]; and whether glucose–insulin trajectories are modified by GLP-1 receptor agonists or SGLT2 inhibitors [98,99,100,101,102,103,104,105]. Such trials must demonstrate a phenotype-by-treatment interaction and clinically meaningful outcomes before response patterns can inform therapy. No clinically actionable score is currently justified.

## 10. Conclusions

Human studies support discrete postprandial phenomena, including transient FMD changes [9,10,11], monocyte activation [32,33,58], soluble endothelial responses [12,65], variable endotoxin-related signals [37,38,39,42,47] and lipid/remnant excursions [15,16,18,19,28,30]. Exercise [44,68,69,89], diet [52,70,71,72,73,94] and lipid-lowering interventions [33,34,35,89,95,96] can modify some individual responses. These observations do not establish a coherent integrated syndrome, greater sensitivity than fasting assessment or incremental prediction of cardiovascular outcomes.

PRISM-CM should therefore be interpreted as an author-developed, hypothesis-generating evidence map comprising five candidate biological domains and cross-domain recovery kinetics. Its illustrative response patterns are not validated endotypes, and no composite score is proposed. Reference ranges, age- and sex-specific norms, repeatability, independent replication, clustering structure, and outcome-linked thresholds remain unknown. Recent trial-level evidence also cautions against inferring clinical benefit from remnant-cholesterol change alone [29]. PRISM-CM is not ready for clinical use or treatment selection; potential future diagnostic and prognostic applications require prospective validation against established comparators.

## Figures and Tables

**Table 1:** Representative human evidence informing candidate PRISM-CM domains and limitations of their integration.

Candidate Domain or Dimension	Representative Human Evidence	Design, Population and Challenge	Evidence-Supported Observation	Qualitative Maturity and Main Limitation
Lipid/remnant burden	Karpe [23]; Bae [11]; Varbo [18]; Hou [30]; Peletier [33]; Byrne [29]	Serial meal studies; prospective cohort (*n* = 107, normal fasting lipids; 1500-kcal, 60–fat OFTT; 10 h sampling; 6-year follow-up); small randomised mechanistic study (*n* = 28, hypertriglyceridaemia).	Postprandial TG/remnant kinetics and ApoB-48 are measurable; selected lipid responses associate with vascular surrogates, and ApoC-III lowering alters lipid and cellular responses.	Moderate evidence for the individual lipid domain. Longitudinal data are small and single-centre; intervention findings use surrogate endpoints; treatment-level evidence does not validate integrated prediction.
Glucose–insulin stress	Esposito [13]; Ceriello [87]; Schwander [77]; Aunbakk [88]	Experimental hyperglycaemia; small, randomised meal studies; 4 h standardised mixed-meal study in 190 adults with abdominal obesity.	Glucose and insulin trajectories vary with phenotype, dose and sex; some acute glycaemic patterns accompany oxidative and endothelial changes.	Established standalone postchallenge axis, but PRISM-CM-specific integration, thresholds and incremental values beyond OGTT/HbA1c/CGM are untested.
Endotoxin handling	Erridge [37]; Ghanim [42]; Clemente-Postigo [47]; Vors [38]; Mo [51]; Laugerette [39]	Mostly small meal-challenge studies, including crossover work in eight lean and eight obese men; independent cohorts of 98 and 62 participants provided contrary assay findings.	Some meals are followed by changes in LPS-related measures tied to fat dose, chylomicrons or adiposity; other studies find values below assay sensitivity or no consistent endotoxaemia.	Low-to-moderate and inconsistent. Assay platform, preanalytics, meal composition and lipoprotein binding materially affect results; prospective outcome value is unknown.
Innate immune-cell activation	Hyson [58]; Gower [32]; Peletier [33]	Small meal-challenge studies, often male-only; randomised mechanistic intervention in 28 participants with hypertriglyceridaemia.	Monocyte surface markers, lipid droplets, monocyte–platelet aggregates and ex vivo adhesion can change after lipid exposure and respond to ApoC-III lowering.	Exploratory. Evidence is largely single-centre; uses labour-intensive surrogate assays and lacks reproducibility and outcome validation.
Endothelial response	Vogel [9]; Plotnick [10]; Nappo [12]; Cortés [72]; Tyldum [68]; Yeboah [63]; Wilson [26]	Small acute crossover/within-person meal studies; FMD cohorts; 2025 systematic review of 16 acute low-carbohydrate, high-fat trials, only two of which assessed endothelial function.	Acute FMD impairment is reported after some high-fat meals and can be modified by exercise or meal composition; soluble markers are less consistent.	Moderate evidence for acute FMD as an individual research measure, but protocols are heterogeneous; operator dependence is substantial and PRISM-CM integration lacks prospective validation.
Cytokines (secondary measurements)	Limani [78]	Double-blind randomised four-way crossover trial; *n* = 18; 706 kcal meals with 40 g fat; 93 inflammatory proteins profiled.	No coherent directional inflammatory-protein signature distinguished the fatty-acid sources.	Low and inconsistent for acute meal responses; multiplicity, timing, assay sensitivity and small samples limit interpretation. Not treated as a separate PRISM-CM module.
Cross-domain recovery kinetics	Herieka [53]; Mora-Rodriguez [89]; de Souza [65]; Aunbakk [88]; Dessing [90]; Dörner [91];	Serial challenge studies with different windows; two-dataset feasibility analysis of inflammatory composites; small repeated-meal time-of-day study (*n* = 8).	Peak timing and return toward baseline vary by biomarker, phenotype and clock time; a minimal cytokine composite did not reproduce across datasets.	Author-proposed analytic dimension only. No validated definition, repeatability threshold, independent prognostic value or evidence that recovery is distinct from peak magnitude.

Abbreviations: ApoB-48, apolipoprotein B-48; CGM, continuous glucose monitoring; FMD, flow-mediated dilation; HbA1c, glycated haemoglobin; LPS, lipopolysaccharide; OFTT, oral fat tolerance test; TG, triglyceride.

**Table 2:** Evidence-supported measurements and author-proposed uses within the hypothesis-generating PRISM-CM framework.

Candidate Domain or Dimension	Measurements Reported in Human Studies	Author-Proposed Use Within PRISM-CM	Current Evidential Status	Main Validation Requirement
Lipid/remnant burden	TG, remnant cholesterol, ApoB-48, ApoC-III; peak, AUC and time to peak [18,19,23,28,33]	Represent nutrient-derived lipoprotein burden and clearance.	Individual domain supported; PRISM-CM-specific thresholds and weighting unvalidated.	Standardised meal, assay harmonisation, repeatability and incremental prediction.
Glucose–insulin stress	Glucose, insulin and C-peptide; peak and AUC [13,82,87]	Represent glycaemic and insulin stress alongside lipid measures.	Standalone axis supported; multimodule integration hypothetical.	Meal-specific norms and incremental value beyond OGTT, HbA1c and CGM.
Endotoxin handling	LPS/endotoxin activity, LBP and sCD14; I-FABP as an adjacent exploratory marker [37,38,39,42,43,47,51]	Characterise a candidate gut-derived inflammatory-response axis.	Exploratory, assay-sensitive and inconsistent.	Preanalytics, assay comparability, replication and exclusion of matrix interference.
Innate immune-cell activation	Monocyte CD11b/CD11c/CD16, lipid droplets, monocyte–platelet aggregates and ex vivo adhesion [32,33,57,58]	Provide a cellular readout of postprandial lipid and inflammatory exposure.	Exploratory; supported mainly by small mechanistic studies.	Multi-centre flow-cytometry standardisation, reproducibility and outcomes.
Endothelial response	FMD and selected soluble adhesion or prothrombotic markers [9,11,12,61,63]	Provide a downstream vascular readout.	FMD is an established research measure; integration into PRISM-CM remains hypothetical.	Operator standardisation, repeated measures and outcome-linked prospective validation.
Cross-domain recovery kinetics	No unique biomarker; return-to-baseline descriptors derived across existing measurements [30,53,82]	Describe delayed normalisation within one or more candidate domains.	Author-proposed analytic construct only.	Mathematical definition, repeatability, independence from peak response and prognostic value.

The third column contains the authors’ conceptual proposals. These uses are not clinical recommendations, validated response patterns or components of an established score. Abbreviations: ApoB-48, apolipoprotein B-48; ApoC-III, apolipoprotein C-III; AUC, area under the curve; CGM, continuous glucose monitoring; FMD, flow-mediated dilation; HbA1c, glycated haemoglobin; I-FABP, intestinal fatty acid-binding protein; LBP, lipopolysaccharide-binding protein; LPS, lipopolysaccharide; OGTT, oral glucose tolerance test; sCD14, soluble CD14; TG, triglyceride.

## Data Availability

No new data were created or analysed in this study.

## Supplementary Materials

The following supporting information can be downloaded at: https://www.mdpi.com/article/10.3390/life16081298/s1. Supplementary Figure S1. Evidence-identification and conceptual-synthesis workflow used for the structured narrative review and author-led construction of PRISM-CM. Database searches covered the period from database inception through to 30 June 2026; one subsequently published contextual review was added during final revision. The figure represents an evidence-mapping workflow rather than a PRISMA flow diagram because stage-specific retrieval and exclusion counts were not prospectively recorded and were not retrospectively estimated. Created in BioRender. Kumric, M. (2026) https://BioRender.com/b1w2ibw.
