# Supplementary material for: Postprandial Inflammatory Stress: A Hypothesis-Generating Framework Linking Metabolic, Immune and Vascular Responses
Source: Life (Basel). 2026 Aug 7;16(8):1298. doi: 10.3390/life16081298 (PMC13514309; doi:10.3390/life16081298)
Supplement: Supplementary file 1 [file life-16-01298-s001.zip › life-4441997-supplementary.pdf]

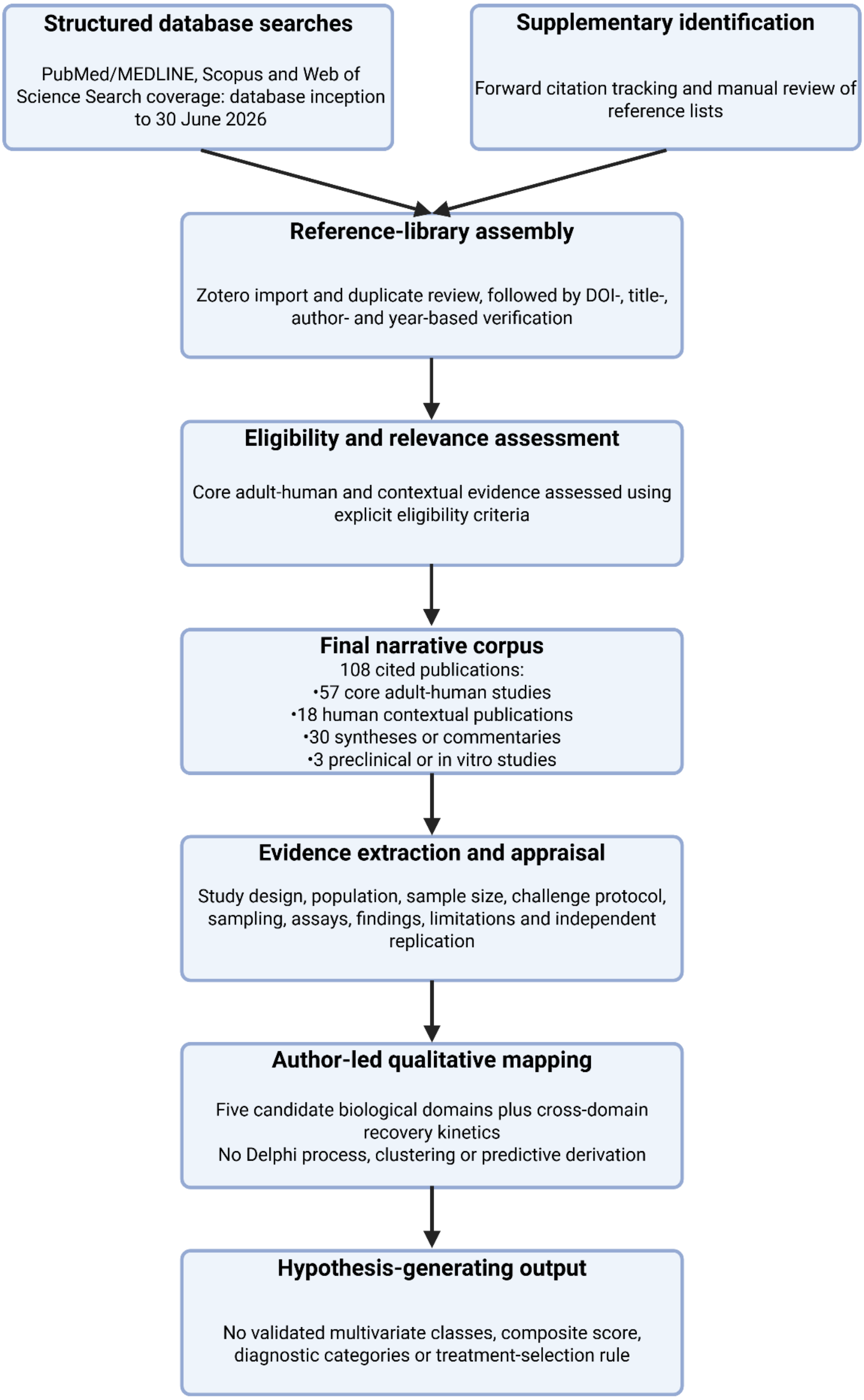

Supplementary Figure S1. Evidence-identification and conceptual-synthesis workflow used for the structured narrative review and author-led construction of PRISM-CM. Database searches covered the period from database inception through to 30 June 2026; one subsequently published contextual review was added during final revision. The figure represents an evidence-mapping workflow rather than a PRISMA flow diagram because stage-specific

retrieval and exclusion counts were not prospectively recorded and were not retrospectively estimated. Created in BioRender. Kumric, M. (2026) https://BioRender.com/b1w2ibw.
